# The cholera outbreak in Syria: a call for urgent actions

**DOI:** 10.1016/j.ijregi.2023.06.005

**Published:** 2023-07-01

**Authors:** MHD Bahaa Aldin Alhaffar, Maria del Mar Moreno Gomez, Jemar Anne Sigua, Anneli Eriksson

**Affiliations:** a Department of Global Public Health, Karolinska Institute, Stockholm, Sweden; b Department of Global Public Health Sciences, Karolinska Institute, Stockholm, Sweden

**Keywords:** Cholera, Outbreak, Syrian crisis, Public health, Earthquake

## Abstract

The ongoing cholera outbreak in Syria poses a significant public health threat that requires immediate and comprehensive attention. The spread of the outbreak is attributed to a combination of factors, including displacement due to armed conflict, chronic water insecurity, inadequate water, sanitation, and hygiene infrastructure, climate change-induced droughts, weakened health system capacity, and political instability. The recent earthquake in the region has further complicated the situation, potentially leading to a surge in cholera cases. The limited capacity of the Syrian health system to handle the cholera outbreak, especially after the earthquake, highlights the urgent need for external support. The political instability in the country has hampered effective responses to the outbreak, contributing to the spread of the disease beyond Syria's borders. It is imperative to prioritize aid to address the fragmented response and provide the necessary resources for comprehensive and effective cholera prevention and control measures. The situation calls for an integrated, multi-sectoral approach that prioritizes economic development, universal access to sustainable safe drinking water, and adequate sanitation. Additionally, community engagement and education are essential for effective disease prevention and control. In conclusion, the ongoing cholera outbreak in Syria is a complex issue that requires urgent attention and action. The combination of armed conflict, water insecurity, climate change, and political instability have contributed to the spread of the disease, further compounded by the recent earthquake. To effectively address the outbreak and prevent its further spread, a comprehensive and integrated approach is needed, with support from the international community.

## Highlights

• The research highlights the current situation of the cholera outbreak in Syria.
• The research reviews the interventions applied by different authorities.
• The research gives recommendations for the future.

## Introduction

The health system in Syria has been severely impacted by years of armed conflict and political instability, resulting in limited access to healthcare services for the population. The system is fragmented, with many healthcare facilities destroyed, damaged, or understaffed, leading to inadequate healthcare delivery, and spread of infectious diseases [1], [2], [3]. Moreover, On February 06, 2023, a catastrophic earthquake struck the regions of southern and central Turkey and northern and western Syria. The earthquake caused massive damage in many cities in Turkey and Syria and resulted in 51.000 casualties in both countries with over 17.9 million affected people, and 195,000 buildings heavily damaged or destroyed [4]. Many health facilities have been reported out-of-service as a result of the earthquake, which increased the pressure on the remaining facilities [5,6].

Although the first cases of cholera were reported in Aleppo in August 2022, it is evident that the outbreak had been spreading for some time before it was officially declared. The government's delay in declaring the outbreak is indicative of broader problems in the health system, including inadequate disease surveillance and response systems, lack of transparent communication, and political interference. Furthermore, the outbreak has now spread beyond the country's borders, underscoring the need for external assistance to help the fragmented response within Syria [7].

The current state of the health system and political landscape in Syria is exacerbating the cholera outbreak, with serious implications for the country and the wider region. The outbreak has highlighted the urgent need for a more coordinated response between local health authorities and international organizations working in Syria. In particular, given the challenges faced after the earthquake, it is essential to strengthen public health processes in North-West Syria (NWS) and provide support to the affected population.

The uprising that led to armed conflict starting in 2011 has had devastating effects on the health system, with over 50% of the country's health facilities destroyed and thousands of healthcare workers have been forced to leave due to direct targeting, insecurity, and protracted conflict [8]. Under these conditions, Syria is now facing a cholera outbreak. Moreover, this situation is controlled by the political context in Syria, as the Syrian government delayed the declaration of the cholera outbreak even though with the increased numbers of diarrheal cases, this factor affects the actual understanding and response to the outbreak and should not be used to gain political advancement. The fragmented nature of the health system in Syria poses significant challenges, including destroyed and understaffed healthcare facilities, limited resources, and a lack of coordinated response. On the other hand, the health system in NWS faces its own set of unique circumstances and challenges. While the armed conflict has also affected NWS, it is important to note that the region operates with different governance and administrative structures, which impact the provision of healthcare services. As a result, approaches to healthcare delivery, resource allocation, and response mechanisms differ between the two regions.

In over a decade of armed conflict in Syria, the spread of infectious diseases, including water-borne diseases such as cholera, has been exacerbated by the lack of access to proper water, sanitation, and hygiene (WASH) due to either physical destruction of established water systems or migration into insufficient and crowded camps [9], [10], [11]. The source of contamination has been associated with the ingestion of untreated water from the Euphrates River in the governorates of Deir-Ez-Zor and Ar-Raqqa. In other governorates, it may have been a result of irrigating plants with tainted water [12,13].

According to the recently published report by World Health Organization (WHO) [14], the cholera outbreak in Syria continues to pose a significant threat to public health, there have been 77,561 suspected cases of acute watery diarrhea reported since August 25, 2022, including 100 attributed deaths, with a case fatality rate of 0.13%. Although the reported number of suspected cases has reduced in some areas, the overall cumulative cases continue to increase. The positivity rate of cholera has been reported as 42.3% in 4454 rapid diagnostic tests, and 868 of 3336 stool samples have tested positive for Vibrio Cholera. The latest epidemiological data show that some areas of NWS (Idelb and north of Aleppo) have reported an increase in cases in some sub-districts, such as Dana, Areha, Aferin, and Azaz [14,15].

The outbreak has also affected internally displaced persons (IDPs) living in camps in NWS and North-East Syria. A total of 6561 suspected cases and seven associated deaths have been reported from IDP camps in these regions. The governorates of Idleb, Deir Ez-Zor, Aleppo, and Raqqa have been particularly affected, with attack rates ranging from 19.7-27.1% [12,15].

The outbreak must not be politicized, and efforts to prevent and control the spread of cholera must remain a top priority for all stakeholders involved. Preventive measures such as the provision of safe water, improved sanitation and hygiene practices, and effective surveillance and response systems must be urgently implemented to prevent further spread of the disease and reduce preventable deaths [5], [6], [7].

The ongoing cholera outbreak in Syria has been exacerbated by the recent catastrophic earthquake, which has further weakened the country's already fragile health system. The earthquake's direct effects on NWS have worsened the outbreak, with public health processes paralyzed for several days. This delay in response has had serious consequences for the affected population, as cholera cases have continued to rise, with external aid, there is an opportunity to support the rebuilding of public health processes in NWS and control the spread of the outbreak.

The aim of this paper is to document the current situation and discuss the underlying factors of the outbreak, particularly in NWS, to better understand the country's health system preparedness and recommend strategies for the future.

### Factors that contributed to the ongoing cholera outbreak in Syria

The underlying factors for the ongoing cholera outbreak in Syria include displacement due to armed conflict situations; chronic water insecurity and lack of WASH infrastructure; droughts due to climate change; and weakened health system capacity [16]. The IDPs due to the armed conflict in Syria are considered the most vulnerable to the cholera outbreak due to their limited access to proper and safe healthcare, WASH, and food [8,^9^,17]. The recent earthquake affected the northwest of Syria, and worsened the WASH situation, as reports before the earthquake showed that around 30% of the country's population depends on the Euphrates River are likely to be drinking contaminated water [17].

### Health system preparedness and response

The preparedness and response of a health system play a crucial role in controlling the spread of diseases like cholera. It is essential to note that in the NWS the health system's preparedness and response to the outbreak is still inadequate due to the ongoing conflict has not only resulted in the physical destruction of health infrastructure but has also led to the criminalization of healthcare workers’ work in some instances, forcing them to leave their positions [8,^18^,19].

The WHO has reported that the number of functioning health facilities in Syria is not sufficient to provide an adequate response to the cholera outbreak, and underreporting of cases is possible due to limitations in testing and reporting capacity [20]. The inadequate surveillance systems in detecting diseases have also been observed in previous studies [7,21]. These limitations, combined with the increase in diarrheal cases even before the official declaration of the cholera outbreak in September, suggest that the outbreak could have been spreading for a long time without detection [13].

Furthermore, the lack of testing capacity and the delay in reporting of cases have made it challenging to understand the full extent of the outbreak and respond appropriately. It is essential to improve the testing and reporting capacity of the health system and strengthen the surveillance systems to detect and respond to outbreaks like cholera promptly. This requires a coordinated effort between local health authorities and international organizations working in Syria.

## Discussion

The recent surge in cholera cases has been a global concern, with 24 countries reporting cases since the beginning of 2023 [22]. This includes African regions such as Malawi, Mozambique, South Africa, Tanzania, Zambia, Zimbabwe, Ethiopia, Kenya, and Somalia, where outbreaks have been reported to be spreading with increasing numbers of reported cases and further spreading to new areas. The situation in the Horn of Africa is especially worrying, with population movement driving transmission across borders [14,22]. The outbreak is also compounded by operational constraints in fragile and conflict-affected states, such as Syria, which challenges the implementation of prompt and adequate responses to disease outbreaks [23]. Moreover, the capacity to respond to multiple and simultaneous outbreaks is strained globally, with shortages of the oral cholera vaccine (OCV) and overstretched public health and medical personnel dealing with multiple disease outbreaks and other health emergencies at the same time [22]. The Global Task Force on Cholera Control launched a global roadmap for the strategic elimination of cholera by 2030, which highlights the need for an integrated multi-sectoral approach to the prevention and treatment of cholera [6]. However, given the current global health situation, such efforts require greater collaboration and resources to address the underlying systemic challenges faced by many countries.

Several factors contribute to the current cholera outbreak in Syria, including displacement, water insecurity, lack of WASH infrastructure, climate change, and weakened health system capacity, with a particular focus on the ongoing armed conflict [24]. WHO's response plan highlights several challenges and gaps, including low community awareness of cholera prevention measures, funding gaps, the limited global supply of the OCV, and lack of institutional knowledge and capacity on cholera [25,26].

It is important to note that the delay in OCV delivery can have significant implications for outbreak control. In the case of Syria, there were delays in the Ministry of Health's request for OCVs from the International Coordinating Group, which led to the vaccines arriving just before an earthquake hit the northwest region [27], Moreover, it is important to mention that Ministry of Health does not operate in the NWS, which further complicate the management of the OCV campaign against the outbreak. Unfortunately, WHO did not provide funding for distribution, leaving Non-Governmental Organizations to bear the cost of running OCV campaigns in NWS after the earthquakes. This highlights the role of politics, weak public health systems, and bureaucracy in responding to outbreaks. The international community should take note of these challenges and work toward more efficient and effective delivery of OCVs, especially given recent challenges in the global supply of OCVs [22]. Despite debates around the effectiveness of OCVs in the medical literature [28], their application during cholera outbreaks in emergency situations such as Syria can help mitigate the impact of outbreaks.

Effective community engagement is critical for the success of any disease prevention and control program, particularly in conflict-affected settings where trust in government and healthcare systems may be low. This is especially important in Syria, where years of conflict have eroded trust in the government and healthcare providers, and vaccine hesitancy is prevalent. Therefore, it is important to work with local communities and leaders to build trust and ensure that accurate information about disease prevention and control measures is communicated clearly, as community engagement seem to be an effective tool in the Syrian community when used for disaster response [29]. Additionally, economic development and universal access to sustainable safe drinking water and adequate sanitation, including the improvement of environmental conditions, the rehabilitation of damaged health facilities, and the improvement of early warning systems should be prioritized [23,30].

Moreover, the devastating earthquake further increased the risk of additional spread of the cases, as many water treatment points were affected, more than 500 thousand became homeless and living in refugee camps, and the health infrastructure is not capable to responded to the increased number of the cases [31,32].

The ongoing conflict, displacement, water insecurity, and weakened health system capacity have contributed to the current outbreak. Urgent actions are needed, including political and governmental support for humanitarian aid access, better WASH interventions, and the prompt delivery of OCVs.

Preventive measures are crucial in halting the spread of cholera in Syria. The main priority must be rebuilding the country's health system and increasing access to safe drinking water and sanitation facilities, particularly in conflict-affected areas, however, this will not be feasible without political stability or serious political change in the governing body in Syria. International organizations should provide technical and financial support to strengthen the country's response, including training and equipping healthcare workers, improving disease surveillance, and expanding access to testing and treatment. It is also critical to ensure that the outbreak response is not manipulated for political gains, as this can undermine the efforts to control and prevent the spread of the disease. All parties involved must prioritize the health and well-being of the Syrian people above political interests.

## Conclusion

The cholera outbreak in Syria is a tragic consequence of the decade-long conflict that has severely impacted the country's health system and hindered its ability to effectively respond to public health crises. The situation has been further compounded by the recent devastating earthquake, which primarily affected the areas where cholera is spreading. This compounding effect has created a more complex and challenging scenario for addressing the outbreak and providing adequate healthcare services to the affected population. Further examination is needed to understand the distinct challenges faced by different regions, such as NWS, within the broader context of the country's health system. The interplay of political factors and the politicization of aid play a major role in the response to this outbreak. It is imperative to prioritize a depoliticized and localized humanitarian aid approach, ensuring transparency and accountability, and strengthening the health system's capacity in NWS, including through collaboration with the health cluster and Syrian NGOs, which can contribute to an effective response. By addressing the underlying issues and implementing a coordinated and robust response, we can strive to prevent similar outbreaks in the future.

## Declarations of competing interest

The authors have no competing interests to declare.
